# Geometry-Induced Capillary Rise and Directional Flow in Porous Lattice Structures

**DOI:** 10.1021/acsami.6c00662

**Published:** 2026-04-06

**Authors:** Yunsan Choi, Josue Yaedalm Son, Hyejeong Kim

**Affiliations:** † School of Mechanical Engineering, 34973 Korea University, Seoul 02841, Republic of Korea; ‡ Max Planck Institute for Dynamics and Self-Organization, Am Faßberg 17, 37077 Göttingen, Germany

**Keywords:** capillary rise, porous structure, body-centered cubic, 3D printing, directional fluid transport

## Abstract

Precise control of capillary-driven liquid transport in porous media underpins numerous interfacial processes in microfluidics, water harvesting, and biomimetic systems. Conventional random porous materials exhibit structural heterogeneity that yields stochastic and irreproducible flow behavior. It was hypothesized that three-dimensional ordered lattices with well-defined geometry, particularly body-centered-cubic (BCC) lattices, could realize deterministic and tunable capillary rise by regulating structural parameters such as the strut diameter, aspect ratio, and unit-cell configuration. To validate this hypothesis, BCC lattices with systematically varied structural parameters were produced by using an additive-manufacturing approach, and capillary rise behavior was examined across geometries. Visualization techniques, including optical- and X-ray-based methods, were used to elucidate the progression of liquid fronts and meniscus evolution. A force-balance model was developed to predict the maximum rise height by incorporating adhesive and gravitational effects within the lattice. Geometric periodicity and asymmetry were found to strongly govern the interfacial transport behavior. Larger strut diameters and denser lattice arrays enhance the capillary height by increasing Laplace pressure and extended liquid–solid contact perimeter. Multicell configurations promoted cooperative meniscus coalescence and triangular wetting fronts, yielding predictable and anisotropic fluid propagation. Moreover, gradient-configured lattices with asymmetric strut distributions yield passive yet directional liquid transport, driven by spatial variations in hydraulic resistance. These findings extend classical capillary theory to ordered three-dimensional porous networks, unveiling geometry as a powerful design parameter for programmable, energy-efficient fluidic, and interfacial systems.

## Graphical abstract

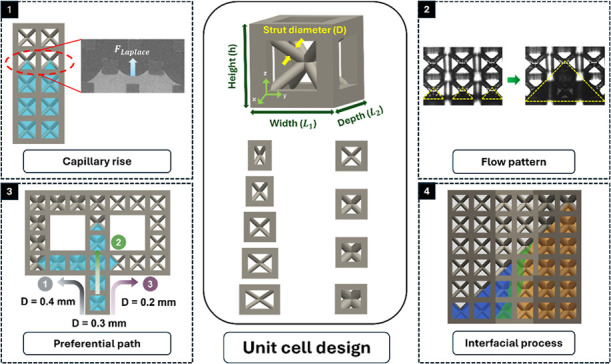

## 1 Introduction

Porous structures, characterized by continuous or discontinuous void spaces within a solid matrix, are essential components in a wide range of fluid-related applications due to their ability to facilitate fluid transport through internal pore networks.^1–3^ Their high specific surface area and tunable permeability make them particularly well-suited for enhancing solid–fluid interactions, thereby enabling efficient mass transport^4,5^ and heat transfer.^6,7^ Owing to these attributes, porous materials have been extensively used in various fields, such as filtration,^8,9^ adsorption processes,^10,11^ catalysis,^12–14^ electrochemical energy storage systems including fuel cells and batteries,^15–17^ biomaterial scaffolds for tissue engineering,^18,19^ and passive cooling systems.^20,21^ These applications highlight the fundamental significance of porous structures in the development of both conventional and emerging fluidic technologies.

Despite these advantages, conventional random porous structures exhibit inherent limitations in precisely predicting and controlling fluid flow, largely due to irregularities in pore size, connectivity, and spatial distribution.^22,23^ Such heterogeneity often leads to biased flow paths, increased pressure losses, and reduced mass transport efficiency, thereby compromising both reproducibility and overall system performance.^24^ Moreover, classical models such as Darcy’s law and the Kozeny–Carman equation are often inadequate for describing the complex behaviors of disordered porous geometries.^25,26^ These challenges underscore the growing need for ordered porous architectures that enable more predictable and controllable fluid transport for advanced applications.

Recent advances in additive manufacturing have enabled the precise fabrication of high-resolution, three-dimensional (3D) ordered porous structures, such as lattice architectures, which were previously difficult to achieve using conventional fabrication methods.^27–29^ This progress has fueled interest in the systematic design and tuning of the fluid transport properties at the microscale. In such porous materials, the unit cell, defined as the smallest repeating element of the lattice, can be precisely engineered by adjusting parameters such as the strut diameter, length, and interconnection angles.^30^ Strut-based lattice architectures, while geometrically more complex than simplified pore or channel models, provide enhanced mechanical stability and structural integrity, which are essential for maintaining open and continuous flow pathways during capillary infiltration. This level of control extends beyond simple modifications of pore size and distribution, allowing for the precise engineering of lattice geometries.^31–33^ Building upon these capabilities, recent studies have demonstrated the ability to predict capillary rise heights and tune fluid-flow behavior by varying the structural parameters, thereby extending the utility of ordered lattices to diverse applications.^34–36^ In this emerging framework, geometry-dependent equilibrium models have been developed in which structural parameters determine the balance between capillary adhesion and gravity, confirming that liquid transport in ordered lattices can be deterministically defined through architectural design. Nonetheless, a systematic framework that quantitatively elucidates and analytically explains the mechanisms of capillary rise from individual unit cells to multicellular arrays remains insufficiently developed.

This study addresses this research gap by systematically investigating the capillary rise in body-centered cubic (BCC) porous lattices, which offer inherent symmetry and geometric periodicity that are advantageous for predictable fluid transport. Through integration of experimental visualization with a theoretically grounded force-balance model, a unified framework is established that connects microscale geometric parameters, including strut diameter, aspect ratio, and unit-cell configuration, to macroscopic transport behaviors such as rise height, velocity, and dynamic wetting front propagation. Extending this analysis from isolated unit cells to multicellular assemblies and gradient lattices further reveals how intentional geometric asymmetry can program directional and highly reproducible liquid transport by creating spatial variations in hydraulic resistance. These results demonstrate the fundamental role of geometry in dictating capillary transport and provide practical design guidelines for energy-efficient passive fluidic systems, ranging from microfluidic devices to moisture-harvesting and bioinspired platforms.

## 2 Materials and Methods

### 2.1 Fabrication of BCC Structures

The BCC structures were designed by using SolidWorks 2023 software (Dassault Systèmes). Each unit cell measured 1.5 mm × 1.5 mm × 1.5 mm with a frame thickness of 0.3 mm (Figure 1). Strut diameters were set to 0.2, 0.3, 0.4, and 0.5 mm. For the aspect ratio models, the strut diameter was fixed at 0.2 mm, and the width-to-height ratios were varied at 1.9/1.5, 1.7/1.5, 1.5/1.5, 1.3/1.5, and 1.1/1.5. All structures were fabricated using a DLP 3D printer (IM2, Carima) printed with a layer stacking thickness of 50 μm, employing Micro 150 resin (CUKH15G, Carima) as the photopolymer material. To remove residual resin, the printed samples were washed by sonication in isopropyl alcohol (Sigma-Aldrich) for 10 min and subsequently cured under ultraviolet (UV) light at a wavelength of 395 nm and a power intensity of 300 W for 30 s using a UV curing system (CL300Pro, Carima). A PVA (Samchun) coating was applied to maintain a stable hydrophilicity during the experimental period. Prior to coating, oxygen plasma treatment (CUTE, Femto Science) was performed at 80 W and 20 sccm of oxygen at 7.6 × 10^2^ Torr for 2 min to improve surface wettability and facilitate interaction with the aqueous PVA solution. Following this treatment, samples were immersed in a 1 wt % PVA solution and placed in a vacuum desiccator (VDC-11, JeioTech) to allow complete infiltration into the internal structure, followed by oven drying at 80 °C for 40 min.

**Figure 1:**
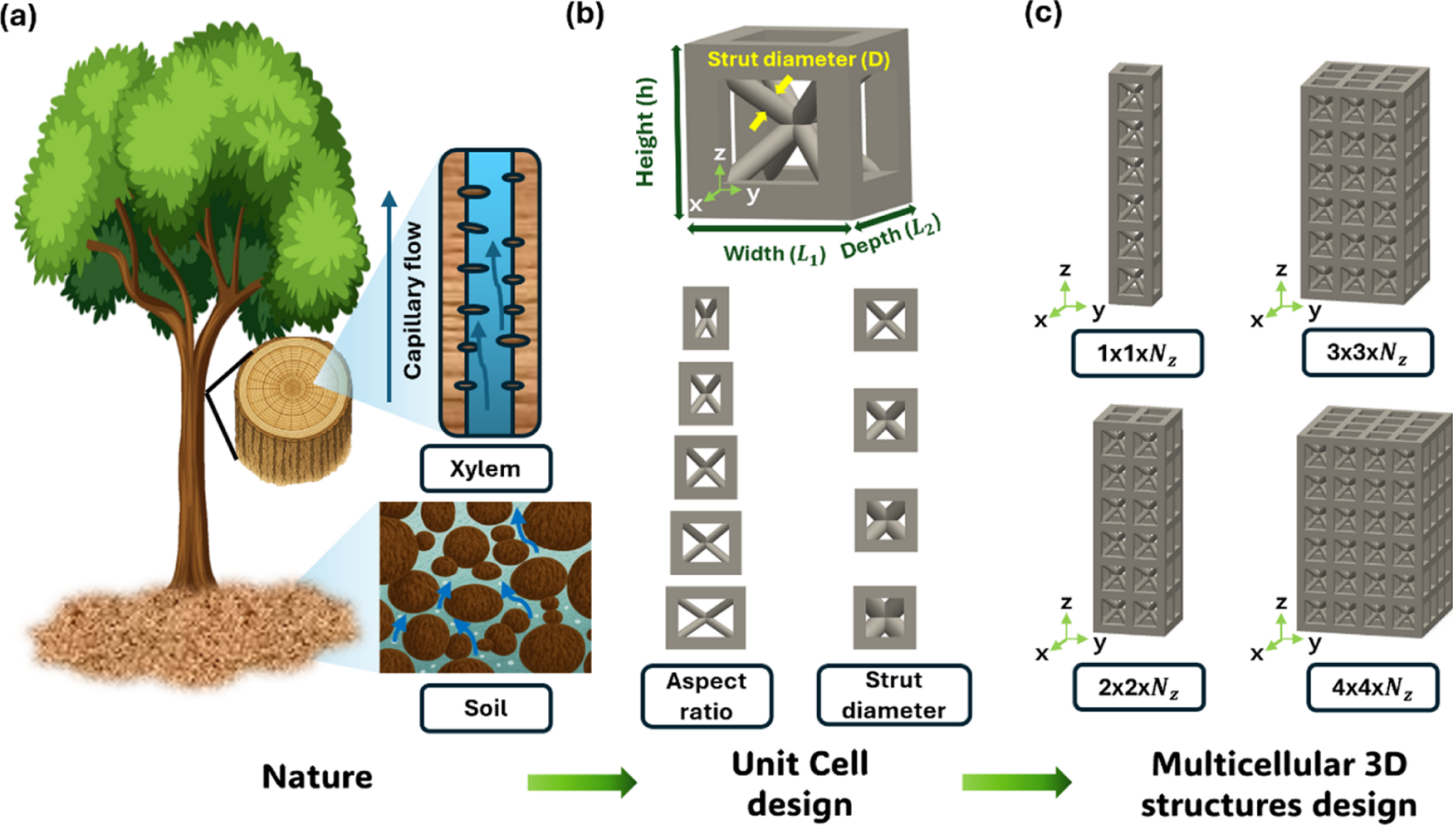
Bioinspired design of a 3D lattice structure. (a) Illustration of natural porous media systems in trees. (b) BCC unit cell design, showing variations in the aspect ratio and strut diameter. (c) Assembly of unit cells into multicellular 3D architectures.

### 2.2 Contact Angle, Surface Tension, and Roughness Measurements

Contact angles were measured using a contact angle goniometer (L2004A1, Ossila) with a 0.5 g/L methylene blue (MB, Sigma-Aldrich) dye solution. Measurements were conducted on both rough and smooth surfaces of the printed samples before and after surface coating. Surface tensions of the methylene blue solution and deionized water were measured with the pendant-drop method using the same instrument. Data analysis was performed by using the Ossila Contact Angle software.

Surface roughness was quantified from SEM images obtained before and after the PVA coating. The images were analyzed using the SurfCharJ plugin in Fiji (ImageJ distribution, National Institutes of Health) to determine the arithmetic mean roughness (*R*_a_) and root-mean-square roughness (*R*_q_). For each condition, multiple regions were analyzed to ensure the statistical reliability.

### 2.3 Morphological Characterization of BCC Structures

The morphological characteristics of BCC structures were examined using a scanning electron microscope (Quanta FEG 250) and a stereoscopic microscope (SZ61, Olympus) equipped with a CMOS camera (KCS3-63S, Korea Lab Tech) utilizing an IMX178 sensor (6.44 M pixels, 1/1.8 in. sensor size). To evaluate the 3D morphology of BCC structures at the point of maximum water rise, synchrotron X-ray microcomputed tomography was conducted at the 6C beamline of the Pohang Accelerator Laboratory. Data were acquired with a 5× objective lens, providing a field of view of 3,328 × 2,808 μm^2^ and a pixel resolution of 1.3 μm/pixel. 3D reconstruction and subsequent analyses were performed using Octopus (inCT) and Dragonfly (Comet) software.

### 2.4 Capillary Rise Experiments

To evaluate the effects of strut thickness, aspect ratios, and unit cell array size (*N*_*x*_ × *N*_*y*_ × *N*_*z*_) on capillary rise, each structure was mounted on a stand positioned above a glass Petri dish containing 50 mL of 0.5 g/L MB (Sigma-Aldrich) solution. The Petri dish was placed on a laboratory jack, and the solution level was gradually raised by vertically adjusting the jack until the bottom surface of the first unit cell was immersed to a fixed depth of approximately 0.3 mm. This procedure standardized the initial contact conditions and meniscus formation. The immersion depth was kept constant for all of the measurements to ensure reproducibility. Following this controlled immersion, the solution was wicked upward through the BCC structure until a stable height was reached. The time-dependent increase was recorded for 30 min using a DSLR camera (D780, Nikon) equipped with an AF Micro NIKKOR 200 mm 1:4D macro lens. Images were captured after equilibrium was reached. Each experiment was repeated five times to ensure statistical reliability. The average capillary rise velocity and average flow rate were defined as the maximum rise height and the corresponding infiltrated liquid volume, respectively, divided by the time required to reach the final plateau height observed within the 30 min recording period. In addition, the average velocity at the unit-cell scale in the early stage was evaluated by dividing the rise height corresponding to the filling of an individual unit cell by the time required for the filling process.

To observe the fluid rise pattern and analyze regional velocity variations within the unit cell, imaging was performed using an inverted microscope (IX73P2F, Olympus) equipped with a 4× objective lens (UPlanFLN, Olympus) and a high-speed camera (Fastcam Mini UX100, Photron) operating at 50 fps. Optical images were captured at a resolution of 1,280 × 1,024 pixels and analyzed using MATLAB (MathWorks) and ImageJ software to quantify the radius of curvature and velocity variations.

### 2.5 Water Uptake Measurement

To compare water uptake between a single-column structure and a multicell array, each sample was mounted on a stand placed above a glass Petri dish containing 100 mL of a 0.5 g/L MB solution. After sufficient time was allowed for the liquid to reach its maximum capillary height, the absorbed water volume was determined by weighing the structures before and after water uptake using a high-precision balance (BCE124i, Sartorius). The theoretical water volume was calculated based on a geometry designed using SolidWorks 2023 (Dassault Systems).

### 2.6 Directional Fluid Transport Analysis in BCC Structures

The influence of structural asymmetry on directional capillary transport was examined by using BCC lattice arrays with varying strut diameters. Two types of vertically gradient structures were fabricated: one with strut diameters increasing from bottom to top (0.2 → 0.3 → 0.4 mm, denoted as BCC_forward) and the other with the reverse configuration (BCC_reverse). Capillary rise experiments were conducted by placing each sample vertically in contact with the MB dye solution, and fluid infiltration behavior was recorded over time using a digital camera (D780, Nikon) equipped with an AF Micro NIKKOR 200 mm 1:4D macro lens. The capillary height and wetting front progression were analyzed by using ImageJ and MATLAB (MathWorks) software.

In addition, symmetric and asymmetric BCC arrays were fabricated with lateral variations in the strut diameter. Methyl orange and MB dye solutions were simultaneously introduced from the opposite sides of the vertically positioned structure at a controlled flow rate of 0.001 mL/min by using syringe pumps (Fusion 4000, Chemyx). The evolution of the liquid–liquid interface and the number of filled unit cells were monitored and quantified at regular time intervals to evaluate interfacial dynamics. All experiments were conducted under ambient conditions, and each configuration was tested in triplicate to ensure reproducibility.

## 3 Results and Discussion

### 3.1 Bioinspired Design and Characterization of Ordered Porous Structures

The natural water transport mechanism in the tree xylem served as inspiration for the lattice design (Figure 1a). In nature, water is efficiently transported through vertically aligned microchannels in the xylem, whereas irregular porous structures in soil create more tortuous flow paths and reduced transport efficiency.^37^ Following this principle, a BCC lattice was designed with well-defined, periodic porous channels to enable directional capillary-driven water transport. It was selected as a representative ordered porous architecture because it offers a regular and systematically controllable geometry, allowing the influence of structural parameters on capillary transport to be examined more clearly than that in random porous media. Moreover, the inclined struts and interconnected pores of the BCC unit cell create multiple possible flow pathways, making this architecture particularly suitable for analyzing both the capillary rise and directional liquid redistribution. The unit cell geometry was defined by key parameters, including the strut diameter (*D*), height (*h*), width (*L*_1_), and depth (*L*_2_), which were systematically varied to control the pore size and wetted surface area (Figure 1b). Two structural modifications were implemented: (i) varying the strut diameter and aspect ratio (*h*/*L*_1_) to regulate the pore scale and fluid transport resistance and (ii) varying the number of unit cells along the horizontal (*x*, *y*) and vertical (*z*) directions to investigate the effect of lattice cell arrangement on water transport behavior. The resulting lattice configurations included single-column structures (1 × 1 × *N*_*z*_) and multicolumn structures (2 × 2 × *N*_*z*_, 3 × 3 × *N*_*z*_, and 4 × 4 × *N*_*z*_), enabling the systematic analysis of capillary rise as a function of structural parameters (Figure 1c).

Various BCC structures were fabricated using a photopolymer resin via digital light processing (DLP) 3D printing and subjected to a hydrophilic surface treatment to facilitate water transport. Owing to the layer-by-layer nature of DLP printing, the unit cells exhibit relatively rough and smooth surfaces. Contact angle measurements were conducted on each surface type to evaluate wettability (Figure S1). Before surface modification, contact angles were 106.3° ± 7.9° on rough surfaces and 91.1° ± 6.7° on smooth surfaces. After oxygen plasma treatment followed by poly­(vinyl alcohol) (PVA) coating, contact angles were significantly reduced to 20.8° ± 4.7° and 41.6° ± 5.1° for rough and smooth surfaces, respectively (Figure S2). The treated surfaces maintained a stable hydrophilicity over time (Figure S3). Notably, the rough surfaces exhibited a lower apparent contact angle after treatment, consistent with classical Wenzel-type wetting, in which surface roughness amplifies intrinsic hydrophilicity and thereby enhances wetting on hydrophilic substrates.^38^ Surface roughness analysis showed no statistically significant change after PVA coating and plasma treatment (Table S1 and Figure S4), indicating that the enhanced wettability primarily originated from surface energy modification rather than geometric alteration. Contact angle hysteresis measurements further revealed a larger hysteresis on rough surfaces compared to smooth surfaces (Table S2). In particular, the reduced receding angle observed on the rough surface suggests stronger contact line pinning associated with surface microtexture, which further supports the Wenzel-type wetting regime.

### 3.2 Effect of Geometrical Parameters on Capillary Rise

To investigate the effect of geometry on capillary rise, BCC structures with strut diameters *D* of 0.2, 0.3, 0.4, and 0.5 mm and aspect ratios *h*/*L*_1_ of 1.5/1.1, 1.5/1.3, 1.5/1.5, 1.5/1.7, and 1.5/1.9 were fabricated in single-column (1 × 1 × *N*_*z*_) and double-column (2 × 2 × *N*_*z*_) configurations. Capillary rise is governed by the balance between capillary forces, which drive the liquid upward, and gravitational forces, which oppose this motion. The interplay of these forces determines both the rising velocity and the maximum attainable height. In accordance with this principle, the capillary rise height and rising velocity increased with larger strut diameters and aspect ratios in both column configurations (Figure 2a,b). This enhancement was attributed to reduced pore size, which decreases the liquid volume subjected to gravitational forces, thereby allowing the liquid to rise to greater heights with higher velocities and flow rates (Figures 2c,d and S5). In the early stages of capillary rise, the differences in velocity among structures were minimal because the gravitational effect was negligible due to the small amount of absorbed liquid (Figure 2e,f). However, as the liquid continued to rise and accumulate, the increasing gravitational force began to counteract the capillary force,^39^ leading to progressively distinct rising velocities depending on the structure. A similar overall trend has been reported for capillary rise in random granular media such as uniform fine sand, where the liquid rises rapidly during the initial stage and then gradually approaches an equilibrium height as gravitational effects become increasingly significant.^40^ In such systems, the maximum rise height is mainly influenced by packing density, whereas in the present BCC lattices, both the rising dynamics and the final rise height are systematically governed by geometric parameters such as strut diameter and aspect ratio. Structures with smaller pores and higher wetted surface areas maintained stronger capillary forces relative to gravity, whereas those with larger pores exhibited a more rapid decrease in rising velocity as gravitational resistance became more dominant (Movie S1).^41^

**Figure 2:**
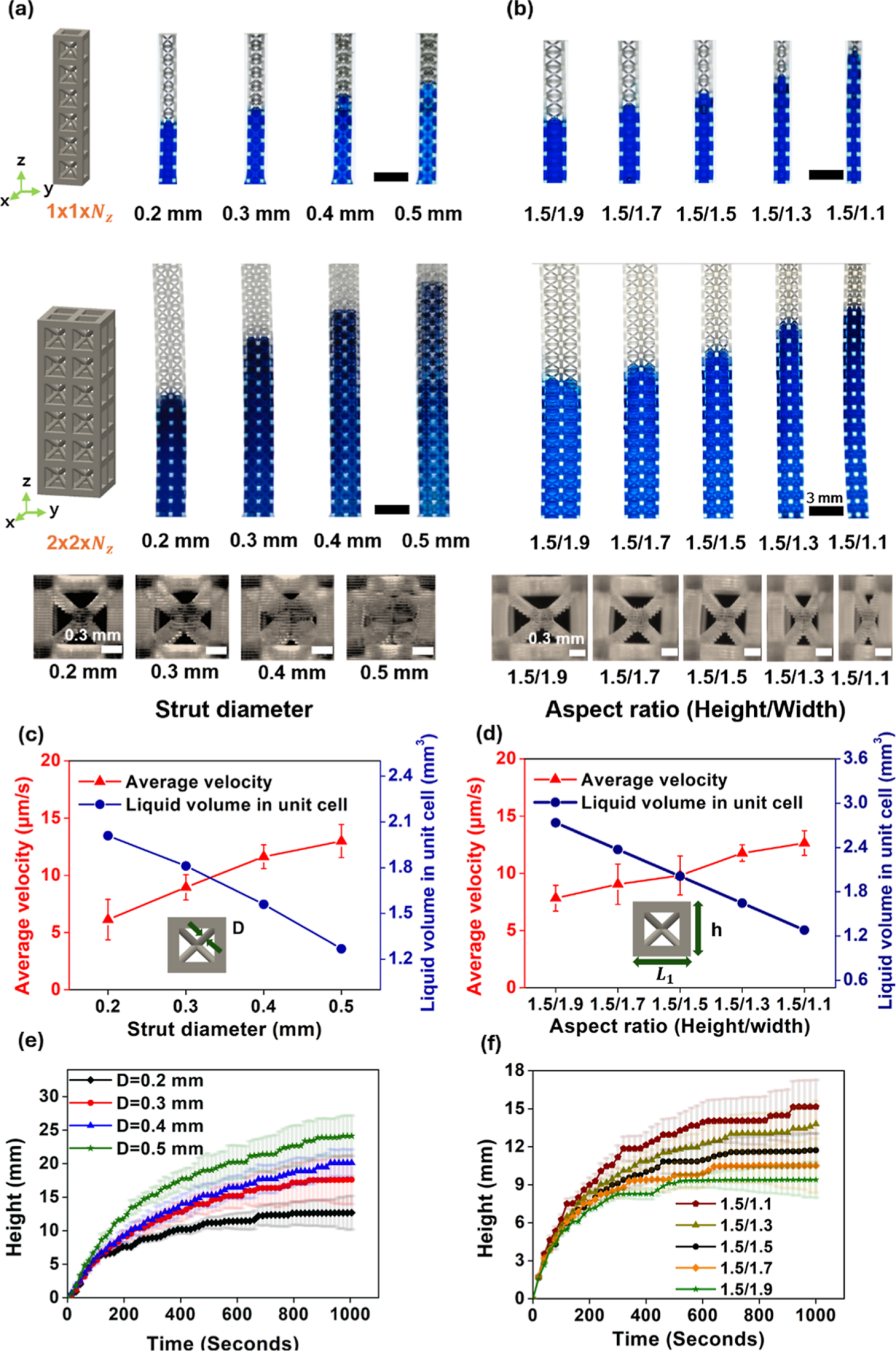
Capillary rise behaviors in BCC lattice structures with varying geometric parameters. Optical images of the unit cells and capillary rise as a function of (a) strut diameter (*D* = 0.2–0.5 mm) and (b) aspect ratio (AR = 1.5/1.1–1.5/1.9) in 1 × 1 × *N*_*z*_ and 2 × 2 × *N*_*z*_ configurations. Average capillary rise velocities and unit cell volume as functions of (c) strut diameter and (d) aspect ratio. Time-dependent capillary rise height in 2 × 2 × *N*_*z*_ lattice structures with varied (e) strut diameters and (f) aspect ratios.

### 3.3 Maximum Capillary Rise Height

Capillary flow is governed by the balance between the upward capillary force generated by surface tension at the liquid–solid interface and the opposing gravitational force acting on the liquid column. The maximum capillary rise height can be estimated using the balance between adhesive and gravitational forces, which is analogous to Jurin’s law.^35,42^ However, Jurin’s law, developed for conventional tubular capillaries assuming a constant liquid–solid contact perimeter, does not directly apply to BCC lattices owing to their periodically varying geometries. In these structures, both the contact perimeter and the resulting adhesive force vary along the vertical axis, necessitating an implicit force balance approach to describe capillary rise behavior.^43–45^

Within each unit cell, the liquid–solid contact perimeter varied periodically, exhibiting local maxima and minima along the vertical direction (Figure 3a). The minimum contact perimeter consistently occurred at the center of the unit cell, indicating a reduced liquid–solid contact perimeter at this position (Figure 3b). Consequently, the maximum capillary height, where adhesive and gravitational forces were balanced, was consistently observed at this position, where the adhesive force was the lowest (Figure 3c). The wetted perimeter at the center was approximated by a circumscribed circle defined by the projected cross section of the diagonal strut (Figure S6). To assess the validity of this approximation, the periodically varying contact perimeter was directly compared to the circumscribed-circle estimate across the investigated geometric range. The relative deviation remained within approximately 1–5%, resulting in a similarly small variation in the predicted equilibrium height (Table S3).

**Figure 3:**
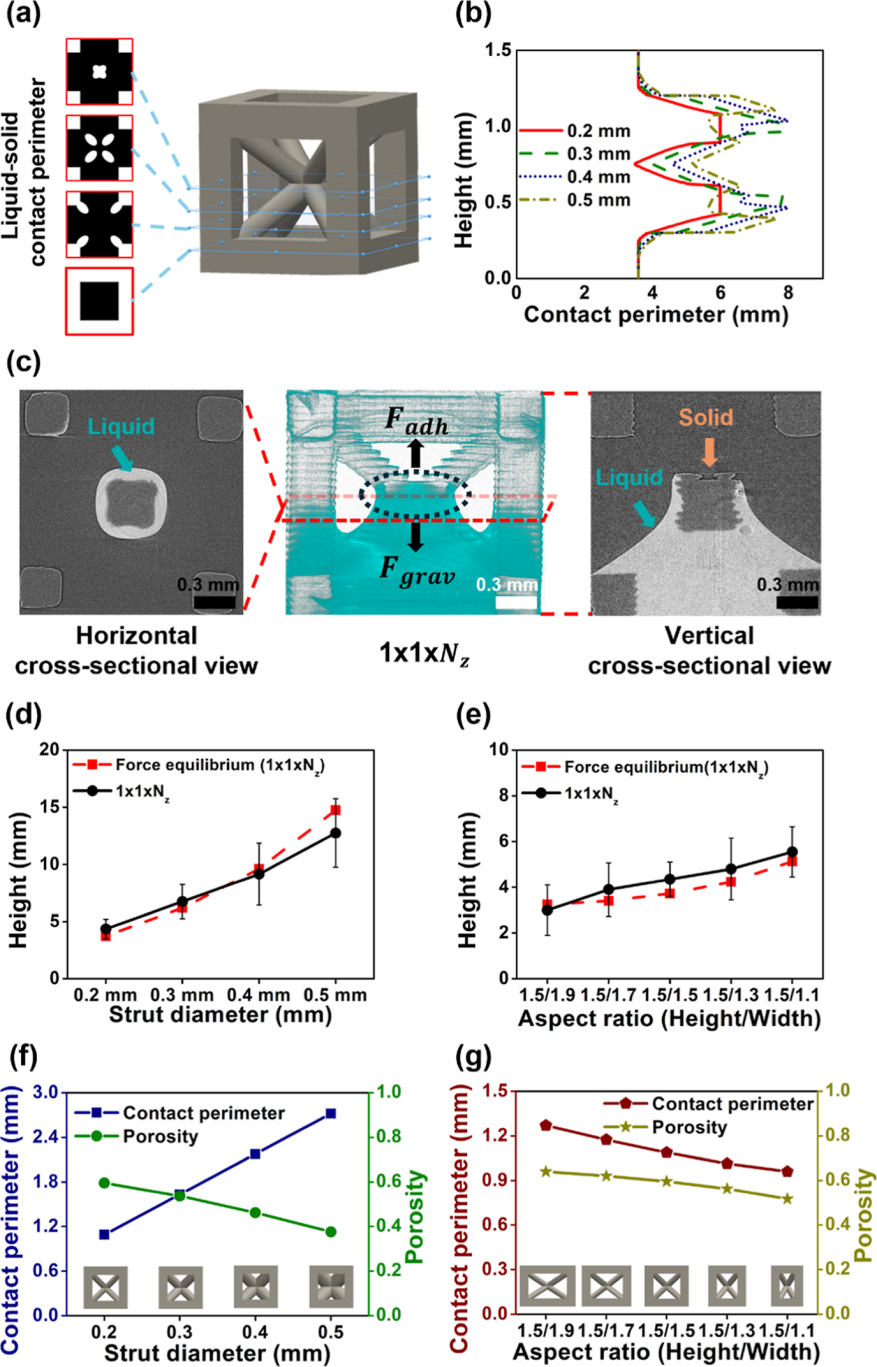
Capillary rise and geometric characteristics of BCC structures. (a) Cross-sectional views of the unit cell showing the liquid–solid contact perimeter at different heights. (b) Contact perimeter as a function of liquid height for different strut diameters (*D* = 0.2–0.5 mm). (c) Synchrotron X-ray image of the capillary rise equilibrium in 1 × 1 × *N*_*z*_ BCC lattices. Comparison of experimental and theoretical force equilibrium-predicted capillary rise heights in BCC lattices (1 × 1 × *N*_*z*_) as functions of (d) the strut diameter and (e) the aspect ratio. Contact perimeter and porosity of BCC unit cells with varying (f) the strut diameter (0.2 mm to 0.5 mm) and (g) the aspect ratio (1.5/1.9 to 1.5/1.1).

The resulting adhesive force is expressed in eq 1 (Supporting Information Method S1) as follows:

1 $${\left\langle {\mathrm{F̅}}_{adh}\right\rangle }_{1\times 1}=\gamma \cos\theta \frac{\pi D}{\sin\alpha }$$

where γ is the surface tension, θ is the contact angle, α is the angle between the diagonal strut and the *x*–*y* plane, and *D* is the strut diameter.

The gravitational force acting on the liquid volume is expressed in eq 2 as follows:

2 $$F_{g}=\rho {ghL}_{1}L_{2}\left(1-\phi \right)$$

where *L*_1_ and *L*_2_ are the width and depth of the unit cell, respectively; ρ is the liquid density; *g* is the gravitational acceleration; *h* is the liquid height; and ϕ is the relative density of the cell, defined as the solid volume fraction (*V*_solid_/*L* ^3^). By applying the force balance between adhesive and gravitational forces (eqs 1 and 2) at the center of the cell, the maximum capillary rise height for a 1 × 1 × *N*_*z*_ is derived using eq 3 as follows:

3 $$h=\frac{\pi D\gamma \cos\theta }{\rho {gL}_{1}L_{2}\left(1-\phi \right)\sin\alpha }$$

This formulation provides an explicit expression for capillary rise in the uniform vertical column of BCC cells. It can also be extended to predict capillary rise in gradient BCC lattice structures (Supporting Information, Method S2). The predicted capillary height for the uniform single-column (1 × 1 × *N*_*z*_) configuration shows good overall agreement with experimental observations (Figure 3d,e). Minor deviations in the exact interface position are expected, as the equilibrium interface does not necessarily coincide precisely with the geometric center of the unit cell. The force balance can be satisfied slightly before or after the minimum-perimeter position. Such minor deviations can arise from the microscale surface roughness of the printed struts, which slightly modifies the effective capillary force without affecting the overall prediction of the maximum rise height. In addition, the present force-balance model is intended as a simplified equilibrium framework and therefore does not explicitly account for local meniscus deformation or slight fabrication-induced geometric variations, which may also contribute to the remaining discrepancies. Nevertheless, the model successfully captures the overall geometry-dependent trend of the capillary rise behavior.

Increasing the strut diameter enlarged the liquid–solid contact perimeter at the center of the unit cell, strengthening the adhesive force (Figure 3f). Simultaneously, the porosity of the structure decreased, reducing the liquid volume subjected to a gravitational force. These combined effects resulted in a greater height difference across samples with varying strut diameters. By contrast, increasing the aspect ratio reduced not only the porosity but also the contact perimeter at the cell center, thereby weakening the adhesive force (Figure 3g). This led to a smaller height difference between structures with different aspect ratios. Overall, simple modulation of the strut diameter and aspect ratio enables precise control of both the contact perimeter and porosity, allowing the maximum rise height and transport velocity to be predictably tuned by the geometry alone.

### 3.4 Capillary Rise in Multicellular Structures

The capillary pressure at the liquid–air interface is described by the Young–Laplace equation (Δ*P* = 2γ/*R*),^46^ where *R* denotes the radius of curvature. Cross-sectional X-ray images of the 2 × 2 × *N*_*z*_ arrays revealed the meniscus formation between adjacent cells. In contrast, single-column structures exhibited no intercellular meniscus formation due to the absence of adjacent unit cells. As a result, wetting was confined to the central region of each unit cell once the liquid reached its maximum height. For the 2 × 2 × *N*_*z*_ arrays, wetting occurred both at the center of individual unit cells and along the shared frames between adjacent unit cells, thereby providing additional adhesive forces (Figure 4a). This extended wetting resulted in a larger water uptake with increasing strut diameter and array size (Figure S7). Furthermore, increasing strut diameter and aspect ratio reduced interstrut spacing, thereby decreasing the radius of curvature (Figures 4b,c and S8). According to the Young–Laplace equation, this reduction increased the Laplace pressure, which amplified the capillary driving force and further enhanced the liquid rise.

**Figure 4:**
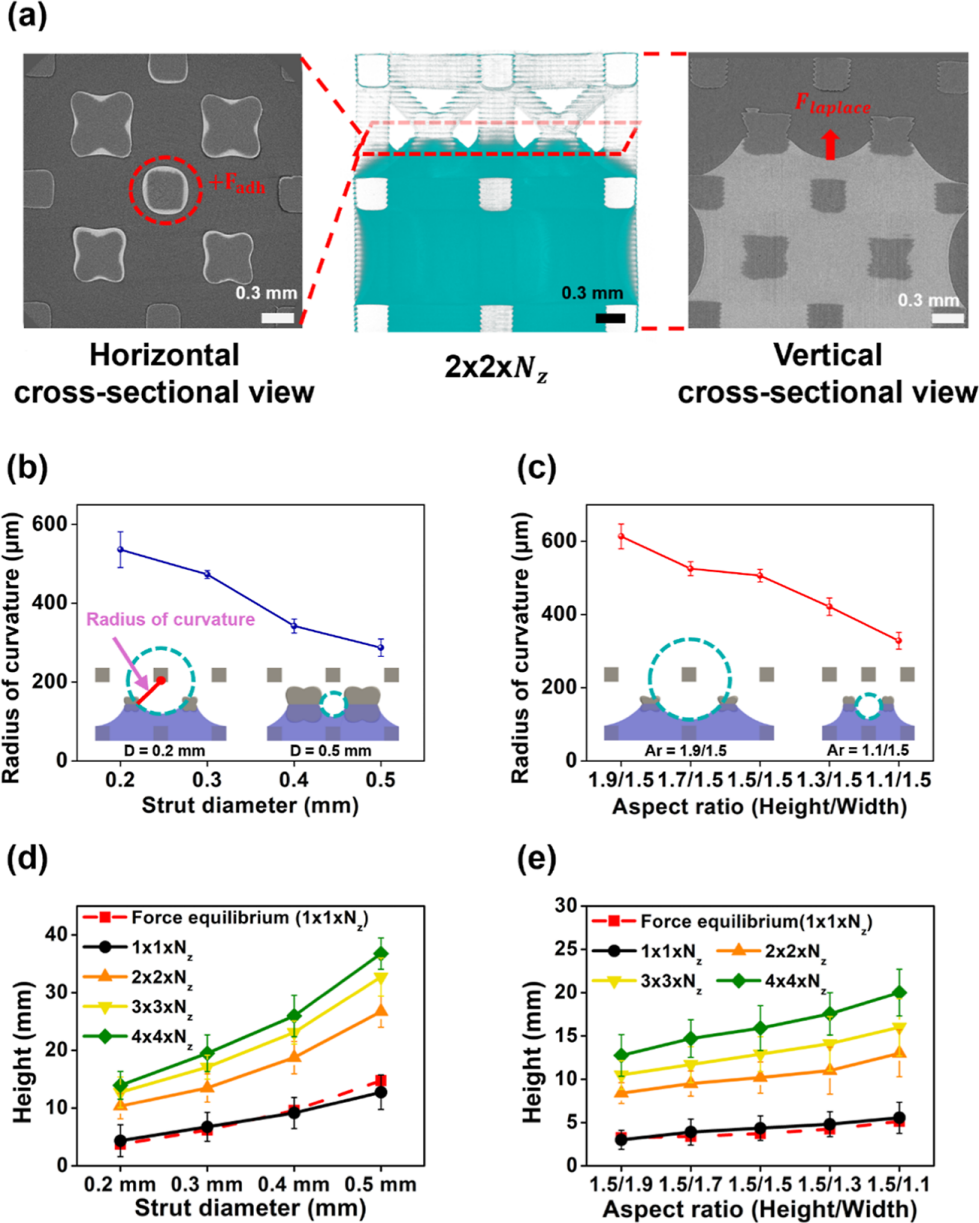
Capillary rise and geometric effect of BCC structures. (a) Synchrotron X-ray images of the fluid interface in 2 × 2 × *N*_*z*_ BCC lattices. Radius of curvature formed at the liquid–air meniscus between adjacent cells as a function of (b) strut diameter and (c) aspect ratio, measured using a 0.05 wt % methylene blue solution. Comparison of experimental and theoretical force equilibrium-predicted capillary rise heights in BCC lattices (1 × 1 × *N*_*z*_ to 4 × 4 × *N*_*z*_) as functions of (d) the strut diameter and (e) the aspect ratio.

Similarly, as the number of cells in the horizontal directions (*N*_*x*_ and *N*_*y*_) increased, the combined effects of Laplace pressure and frame wetting increased the capillary height (Figure 4d,e). As the array size increases, the number of intercellular contact regions grows more rapidly than that of the outer boundary regions, leading to an increasing inner-to-outer interaction ratio (Table S4 and Figure S9). This geometric scaling indicates that cooperative meniscus formation becomes progressively more significant within the interior region, thereby increasing the effective capillary driving force. However, as the array expands, the total absorbed liquid volume also increases, resulting in a proportional rise in gravitational force. When the additional capillary contribution arising from interior cooperativity becomes comparable to the accumulated gravitational force, further enlargement of the array no longer produces a proportional increase in capillary height, leading to the observed saturation behavior (Figure S10). This behavior underscores that capillary transport in ordered lattices is not solely governed by the properties of individual unit cells; rather, the structural complexity that emerges when multiple cells are interconnected shapes the resulting meniscus morphology, enabling controlled modulation of the capillary rise height.

### 3.5 Analysis of the Capillary Rise Pattern Based on Structural Parameters

Capillary rise in BCC lattices exhibited a stepwise pattern (Figure 2e,f), which prompted a detailed visualization of how water filled the interior of the unit cell to clarify the underlying mechanism. During the rising process in multicellular arrays, the central region was filled first, followed by the gradual wetting of the surrounding cells (Figure 5a and Movie S2). Initially, the liquid contacted the bottom strut and rose in a triangular pattern (Figure S11). It then advanced along the frames connecting the bottom adjacent cells, subsequently filling the side regions and, finally, the upper region in a repeating sequence. At the same vertical height (constant *N*_*z*_), the inner cells filled more readily owing to the geometric connectivity and extended wetting interfaces with the liquid already present in adjacent cells. By contrast, outer cells rose more slowly, as they lacked direct contact with prewetted surfaces. These outer regions were not wetted directly from the bottom but instead received liquid laterally from the already filled inner cells. Consequently, this difference in filling behavior resulted in a rise pattern in which the central region was filled first, followed by the gradual filling of the periphery, forming a triangular front during the liquid ascent. Such a geometrically repeated triangular rise pattern contrasts with the behavior typically observed in random porous media, including paper-based fibrous networks, packed granular materials, and polymer foams or sponges, where the advancing wet front develops an irregular and spatially heterogeneous morphology due to stochastic pore connectivity.^47–49^ In those systems, the local rise pattern is generally nonuniform and difficult to predict, reflecting the inherent structural disorder of the pore network. In contrast, the ordered BCC architecture enforces a deterministic filling sequence governed by the unit-cell geometry, leading to reproducible front evolution.

**Figure 5:**
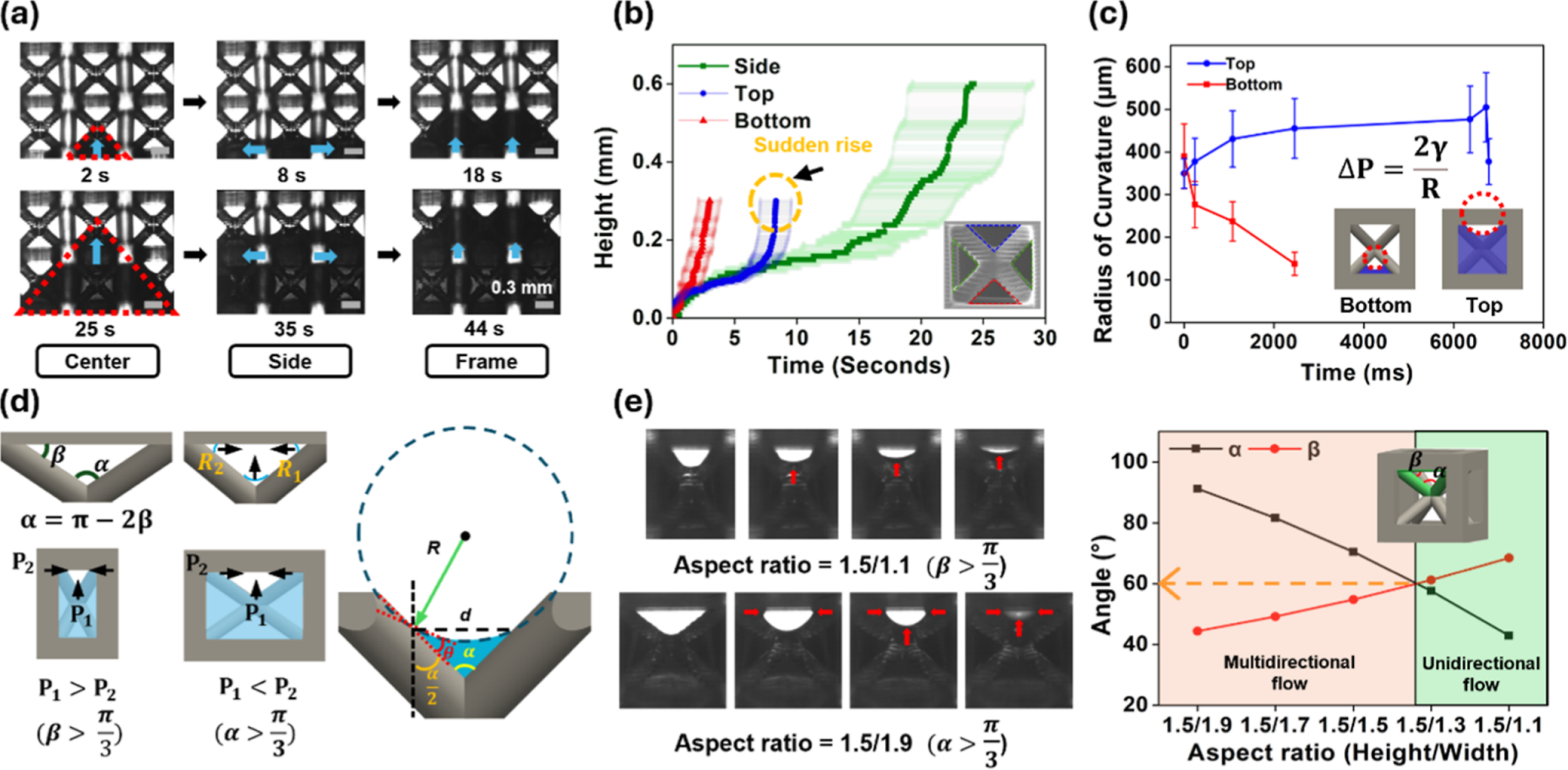
Capillary rise pattern based on structural characteristics. (a) Optical images showing the characteristic capillary rise pattern in BCC structures. (b) Time-dependent capillary rise height measured at three regions: bottom, side, and top. (c) Time-dependent radius of curvature measured at the top and bottom interfaces during capillary rise. (d) Schematic illustrating how Laplace pressure at the top region varies with the curvature angle α. (e) Optical images and the corresponding plot showing how variations in the relative values of α and β influence capillary flow patterns.

Further observations within a single unit cell revealed that liquid filling accelerated noticeably in the bottom region (Figures 5b and S12). This acceleration was caused by a reduced radius of curvature as the liquid rose, leading to an increase in the Laplace pressure (Figure 5c). The narrowing geometry reduced the radius of curvature of the liquid–air interface, thereby increasing the Laplace pressure and further enhancing the rise velocity.^50,51^ Conversely, the upper region exhibited a slower rise because the geometry widened upward, leading to an increased radius of curvature and a corresponding decrease in Laplace pressure. Notably, a sudden acceleration was observed near the top of the structure. Unlike the bottom regions, the top region can receive liquid from both the upward pathway and laterally prewetted side surfaces, effectively introducing multiple filling routes within the unit cell. As the rising front encounters these prewetted regions, capillary bridging is established, leading to a transient acceleration of the upward motion. At this stage, capillary bridging between adjacent wetted regions enabled continuous liquid pathways, facilitating rapid filling driven by enhanced Laplace pressure.^52,53^ This bridging event locally reconfigured the flow-network connectivity and redistributed the pressure field, contributing to the transient acceleration observed in the top region.

Building upon the previous observations of geometry-dependent fluid transport, the influence of specific geometric parameters of the BCC unit cell was analyzed. In particular, the capillary rise was analyzed with respect to two key parameters: α, the angle between intersecting struts, and β, the angle between the strut and the top frame.

The angles α and β are dependent on the aspect ratio, with their relationship defined as α = π – 2β (Figure 5d). After the bottom and side regions were filled, liquid advanced into the top region via multiple paths with the meniscus shape and flow direction influenced by α and β values. The radius of curvature and the corresponding Laplace pressure during the vertical rise can be expressed using eqs 4 and 5 as follows:^54^

4 $$R=\frac{d}{2\cos\left(\theta +\frac{\alpha }{2}\right)}$$

5 $$\Delta P=\frac{2\gamma }{R}=\frac{4\gamma \cos\left(\theta +\frac{\alpha }{2}\right)}{d}$$

where γ is the surface tension and θ is the liquid–solid contact angle. When the aspect ratio was large (i.e., $\beta >\frac{\pi }{3}$ , which arises from the condition β > α = π – 2β), the liquid exhibited predominantly unidirectional upward flow. Here, unidirectional upward flow refers to a flow regime in which the liquid front advances predominantly along the vertical direction with minimal lateral spreading within the unit cell. This behavior was attributed to the narrower vertical spacing and smaller radius of curvature in the vertical direction (*R*_1_), which produced a Laplace pressure higher than that in the lateral direction (*R*_2_), resulting in a faster rise. By contrast, when the aspect ratio was small (i.e., $\alpha >\frac{\pi }{3}$ , which arises from the condition $\alpha >\beta =\frac{\pi -\alpha }{2}$ ), the liquid displayed multidirectional spreading, moving both vertically and laterally (Figure 5e and Movie S3).

In this case, the vertical path required a larger volume of liquid and exhibited an increased radius of curvature, resulting in a lower Laplace pressure. Consequently, the vertical rise was slower and the liquid preferentially spread along the side surfaces before expanding into the top region. While the pore size and volume vary simultaneously with the aspect ratio, the directional preference of capillary rise can be interpreted in terms of the anisotropic Laplace pressure associated with α and β. In this regard, α and β provide a useful framework for describing and rationalizing the directionality of fluid transport. These results highlight that the angles formed by the structural elements and the aspect ratio significantly influence fluid flow behavior; therefore, they can serve as critical design parameters for directionally controlled flow systems.

### 3.6 Directional Fluid Transport in BCC Structures

To investigate the effect of geometric asymmetry on the direction of capillary flow, two gradient-configured BCC lattice structures were designed in which the strut diameter varied in opposite vertical directions. One structure (BCC_reverse) featured a gradual decrease in the strut diameter from bottom to top (*D* = 0.4 → 0.3 → 0.2 mm), while the other (BCC_forward) exhibited an increasing strut diameter along the same axis. The capillary rise behaviors in both structures were experimentally compared to assess the influence of the strut gradient on fluid propagation.

As shown in Figure 5a, the BCC_reverse structure exhibited a faster initial capillary rise than did the BCC_forward structure (Movie S4). This was attributed to the relatively larger strut diameter located in the lower region of BCC_reverse, which reduced the porosity and liquid volume required for the initial filling. Consequently, a stronger Laplace pressure was generated, promoting a rapid capillary rise during the early stage. In this stage, the rise dynamics are primarily governed by the local capillary driving pressure determined by the bottom geometry, while gravity contributes only as a hydrostatic resistance term that remains small at low heights. However, as the liquid continued to ascend, the decreasing strut diameter and increasing porosity toward the top required a larger liquid volume to fill the structure. Simultaneously, the radius of curvature of the meniscus increased, reducing the Laplace pressure and decelerating the rise in velocity. By contrast, BCC_forward began with smaller strut diameters at the bottom, resulting in a higher porosity and weaker Laplace pressure, thus leading to a slower initial rise. As the liquid moved upward, increasing the strut diameter reduced the porosity and volume required for filling, thereby enhancing the Laplace pressure and accelerating capillary rise. Because the radius of curvature progressively decreases along the rise direction in this configuration, the capillary driving pressure increases cumulatively, leading to acceleration despite the presence of gravitational resistance. As a result of this cumulative effect, BCC_forward eventually reached a greater capillary height than BCC_reverse after sufficient time. These results show that even the ordering of the geometric gradient alone can modulate rise dynamics and control flow speed.

Additionally, we observed preferential infiltration into regions with higher structural densities (Figure 6b and Movie S4). In this configuration, the central cell featured a strut diameter of 0.3 mm, whereas the left and right cells were composed of larger (0.4 mm) and smaller (0.2 mm) struts, respectively. Over time, the liquid initially entered the left-side cell with the highest structural density (100 s), followed by a gradual progression into the central cell (130 s) and finally into the less dense right-side cell (450 s). This directional flow can be attributed to the continuity of the liquid phase and preferential wetting along the initial flow pathways, which favor transport into regions with lower hydraulic resistance.

**Figure 6:**
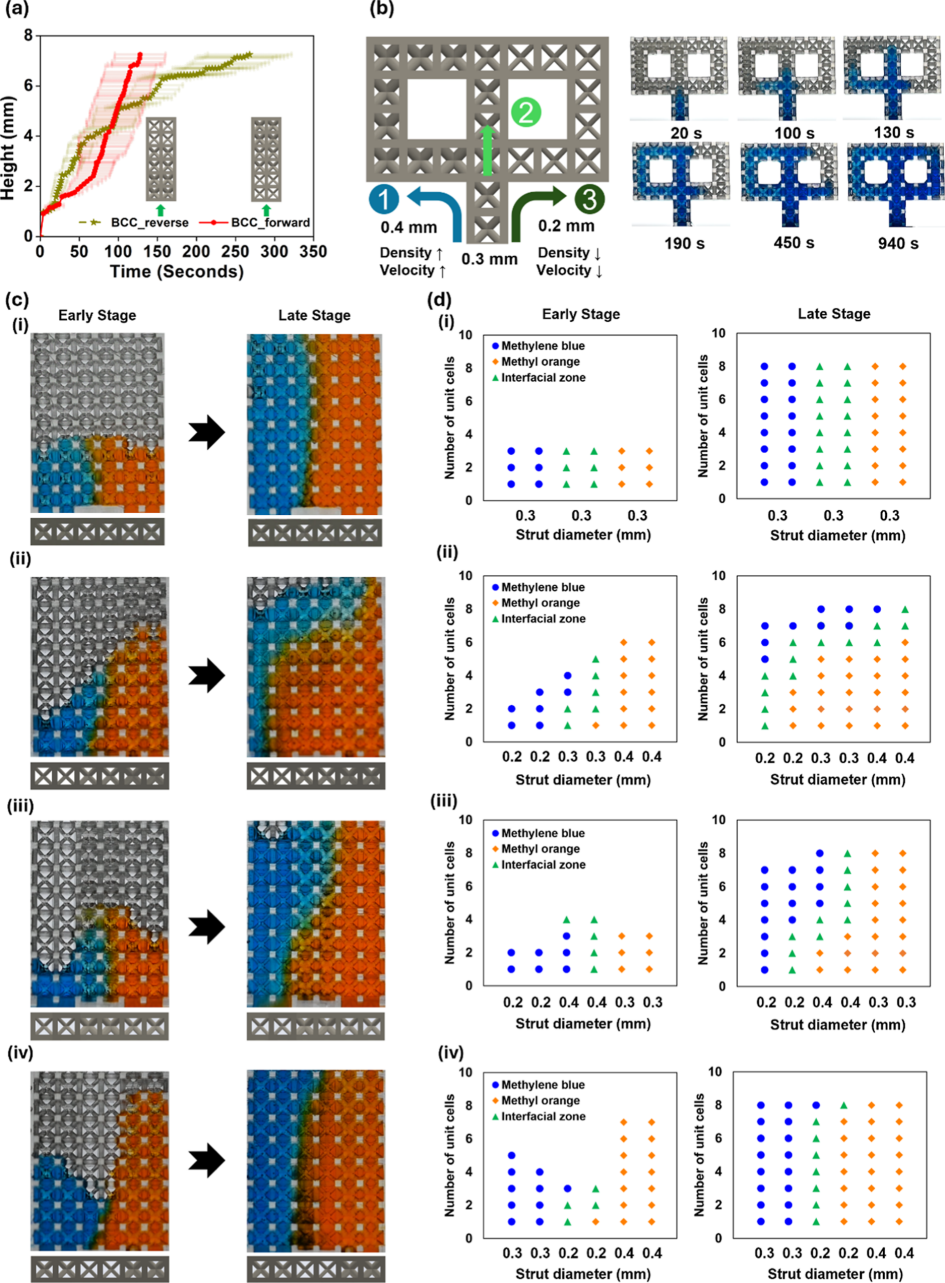
Directional fluid transport in BCC lattices. (a) Capillary rise height over time in BCC_forward and BCC_reverse structures. (b) Optical images showing preferential capillary flow pathways induced by density differences. (c) Optical images and (d) quantitative plots of diffusion boundary layer development in symmetric and asymmetric lattice structures: (i) symmetric (uniform 0.3 mm struts), (ii) asymmetric (0.2–0.3–0.4 mm), (iii) asymmetric (0.2–0.4–0.3 mm), and (iv) asymmetric (0.3–0.2–0.4 mm).

To evaluate the influence of lattice symmetry on interfacial fluid transport, two types of lattice structures were designed: (i) symmetric configuration with identical strut diameters and (ii–iv) asymmetric configurations with varying strut diameters (Figure 6c and Movie S5). In both configurations, methyl orange and methylene blue dye solutions were simultaneously injected from opposite sides at a constant flow rate of 0.001 mL/min to observe the interfacial behavior as the two liquids converged (Figure S13). The early stage corresponds to the regime in which gravitational effects are minimal and capillary rise is dominant, whereas the late stage represents the regime in which gravitational resistance increases and injection-driven rise becomes predominant. In the symmetric structure, a uniform capillary rise was observed in both directions, and the resulting interface remained vertically aligned (Figure 6c­(i)). By contrast, in the asymmetric gradient structure, capillary rise velocities differed depending on the local strut diameter, resulting in a progressively tilted and asymmetric interface that expanded toward the right (Figure 6c­(ii–iv)). This interfacial propagation was further quantitatively analyzed by tracking the number of filled cells and the position of the liquid–liquid interface over time (Figure 6d­(i–iv)). In the asymmetric gradient configuration (Figure 6d­(ii–iv)), the interface gradually shifted from the region with larger struts (low porosity) toward the region with smaller struts (high porosity) as time progressed. This behavior reflects the significant influence of gravity: as the liquid accumulates, rather than continuing upward against the growing gravitational resistance, the liquid spreads laterally into neighboring cells with relatively lower hydraulic resistance and higher permeability. Regions with larger strut diameters exhibited greater internal resistance and required more energy to pass through. As a result, the fluid preferentially moved toward directions with reduced resistance and energy demand.^55–57^ A greater density difference between the lateral ends (e.g., 0.4 mm vs 0.2 mm) produced a more noticeable shift of the interfacial zone toward the lower-resistance side (Figure 6d­(ii)). In contrast, when the cells at both lateral ends were 0.4 and 0.3 mm and the low-resistance unit cell (0.2 mm) was positioned at the center, the interfacial zone remained relatively centered, showing minimal displacement (Figure 6d­(iv)). To further examine whether distinct transport regimes emerge depending on the imposed advection rate, additional experiments were conducted at higher injection flow rates of 0.003 and 0.01 mL/min (Figure S14). At 0.001 mL/min, the transport remained stable and primarily governed by capillary uptake under gravitational influence and no leakage was observed. However, as the injection rate increased, the externally imposed injection pressure progressively exceeded the intrinsic absorption capacity of the lattice, leading to fluid leakage at the lower boundary of the structure. The onset of leakage occurred earlier at 0.01 mL/min than at 0.003 mL/min, indicating a transition from a capillary-dominated regime to an injection-dominated regime. Importantly, despite this rate-dependent transition, the preferential migration of the liquid toward geometrically lower-resistance pathways was consistently observed across all flow rates, confirming that lattice symmetry dictates the directional bias of interfacial transport while the injection rate governs the stability and regime of propagation.

Overall, these findings demonstrate that the flow rate, transport pathway, and interface morphology can be effectively controlled by tailoring the geometric arrangement of the BCC structures. By tuning the spatial distribution of strut diameters via vertical gradients, lateral asymmetry, or array-level configurations, the capillary driving force and hydraulic resistance can be precisely modulated. This strategy enables the passive regulation of fluid transport without external driving forces and provides practical design guidelines for advanced fluidic systems requiring directional or rate-controlled flow.

## 4 Conclusion

On the basis of previous studies on capillarity in random and ordered porous media,^26,58–63^ this study establishes a geometry-based framework for controlling capillary-driven liquid transport in three-dimensional ordered lattices. Through systematic variation of geometric parameters, including the strut diameter, aspect ratio, and unit-cell configuration, it was demonstrated that structural parameters govern capillary height, rise velocity, and wetting dynamics. The simple force-equilibrium model, supported by X-ray visualization, revealed that the enhanced capillary height in multicell lattices arises from intercellular meniscus formation and cooperative wetting along shared frames. These effects amplify the local Laplace pressure and adhesive forces, increasing the equilibrium capillary height beyond single-cell predictions and extending the classical capillary theory to interconnected three-dimensional porous networks.

Moreover, multicellular and gradient configurations revealed directional and programmable fluid propagation driven by meniscus bridging and structural asymmetry, where spatial variations in the Laplace pressure and permeability dictate flow directionality. These findings provide new physicochemical insight into the coupling between Laplace pressure and lattice geometry, advancing the predictive design of interfacial transport beyond conventional surface or material-based strategies.

Looking ahead, the integration of hierarchical, multimaterial, or stimuli-responsive components into these architectures could enable dynamic regulation of capillary flow. In particular, incorporating conductive or functional materials into such geometrically programmable lattices would allow external stimuli to modulate interfacial properties, thereby tuning the flow velocity and pathway preference. Beyond theoretical modeling, this geometric framework provides a scalable design principle for real-world applications. For instance, these lattices can serve as energy-free pumping units in microfluidics, where meniscus bridging overcomes conventional flux limitations. More broadly, the geometry-induced regulation of liquid transport can be extended to systems where controlled fluid supply is critical, such as solar evaporation processes, where it can support stable liquid replenishment and effective alt management. Ultimately, such structural interfacial control offers a versatile toolkit for next-generation energy and environmental technologies.

## Supplementary Material













## Data Availability

Data will be made available on request.

## Notes

Capillary rise in the BCC lattice structures (Movie S1) (MP4)
Capillary rise pattern in the BCC structure (Movie S2) (MP4)
Flow direction based on the relationship between α and β (Movie S3) (MP4)
Preferential capillary flow pathways (Movie S4) (MP4)
Diffusion boundary layer development in symmetric and asymmetric lattice structures (Movie S5) (MP4)
Derivation of the adhesive force for capillary rise in BCC lattice structures (Method S1); extension of the force-balance model to gradient BCC lattice structures (Method S2); surface roughness parameters before and after surface treatment (Table S1); contact angle hysteresis of smooth and rough printed surfaces (Table S2); comparison of approximated and actual wetted perimeters (Table S3); intercellular contact regions and outer boundary regions in *n* × *n* lattices (Table S4); optical images of a BCC lattice structure indicating smooth and rough surfaces (Figure S1); contact angles on bare and coated rough surfaces (Figure S2); time-dependent contact angles variations after surface treatments (Figure S3); SEM images of the BCC structure (Figure S4); average capillary-driven flow rates as functions of strut diameter and aspect ratio (Figure S5); geometric model for evaluating the wetted perimeter at the unit-cell center (Figure S6); water volume as a function of strut diameter for different BCC lattices (Figure S7); 3D synchrotron X-ray images of the radius of curvature in 2 × 2 × *N*_*z*_ BCC lattices (Figure S8); schematic illustration of intercellular and boundary regions in *n* × *n* lattices (Figure S9); maximum capillary rise height as a function of array size (Figure S10); triangular liquid rise patterns in BCC lattices (Figure S11); average capillary rise velocity at different unit-cell positions (Figure S12); experimental setup for visualizing the interfacial zone (Figure S13); and effect of injection flow rate on interfacial propagation and leakage behavior (Figure S14) (PDF)
