# Supplementary material for: Geometry-Induced Capillary Rise and Directional Flow in Porous Lattice Structures
Source: ACS Appl Mater Interfaces. 2026 Apr 6;18(14):21212–24. doi: 10.1021/acsami.6c00662 (PMC13088047; doi:10.1021/acsami.6c00662)
Supplement: Supplementary file 6 [file am6c00662_si_006.pdf]

[Supporting Information]

Geometry-induced capillary rise and directional flow in porous lattice structures

Yunsan Choi[1], Josue Yaedalm Son[1], and Hyejeong Kim[1,2,†]

[1] School of Mechanical Engineering, Korea University, Seoul 02841, Republic of Korea

[2] Max Planck Institute for Dynamics and Self-Organization, Am Faßberg 17, 37077 Göttingen, Germany

†Corresponding author

e-mail: h_kim@korea.ac.kr

phone: +82-2-3290-3379

Keywords: Capillary rise, Porous structure, Body-Centered Cubic, 3D printing, directional fluid transport

Contents of the Supporting Information

Method S1. Derivation of the adhesive force for capillary rise in BCC lattice structures

Method S2. Extension of force-balance model to gradient BCC lattice structures

Table S1. Surface roughness parameters before and after surface treatment.

Table S2. Contact angle hysteresis of smooth and rough printed surfaces.

Table S3. Comparison of approximated and actual wetted perimeters.

Table S4. Intercellular contact regions and outer boundary regions in n × n lattices.

Figure S1. Optical images of a BCC lattice structure indicating smooth and rough surfaces.

Figure S2. Contact angles on bare and coated rough surfaces.

Figure S3. Time-dependent contact angles variations after surface treatments.

Figure S4. SEM images of the BCC structure.

Figure S5. Average capillary-driven flow rates as functions of strut diameter and aspect ratio.

Figure S6. Geometric model for evaluating the wetted perimeter at the unit-cell center.

Figure S7. Water volume as a function of strut diameter for different BCC lattices

Figure S8. 3D synchrotron X-ray images of the radius of curvature in $2\times2\times N_z$ BCC lattices.

Figure S9. Schematic illustration of intercellular and boundary regions in n × n lattices.

Figure S10. Maximum capillary rise height as a function of array size.

Figure S11. Triangular liquid rise patterns in BCC lattices.

Figure S12. Average capillary rise velocity at different unit-cell positions.

Figure S13. Experimental setup for visualizing the interfacial zone.

Figure S14. Effect of injection flow rate on interfacial propagation and leakage behavior.

Movie S1. Capillary rise in the BCC lattice structures

Movie S2. Capillary rise pattern in the BCC structure

Movie S3. Flow direction based on the relationship between α and β

Movie S4. Preferential capillary flow pathways

Movie S5. Diffusion boundary layer development in symmetric and asymmetric lattice structures

Method S1. Derivation of the adhesive force for capillary rise in BCC lattice structures

Based on the Equations (1) from the main text of this paper, the adhesive forces of the BCC structure were theoretically derived. In conventional tubular capillaries, the adhesion and gravity forces acting on the wetted capillary are:

$$F_{adh} = \gamma \cos\theta \int ds = \gamma \cos\theta 2\pi r$$

$$F_g = \rho g h \pi r^2$$

where, γ is the surface tension, θ is the contact angle, s is the liquid-solid contact perimeter, r is the tube radius, ρ is the liquid density, g is the gravitational acceleration, and h is the liquid height. The liquid rises until the adhesive and gravitational forces reach equilibrium. By setting $F_{adh} = F_g$ and solving for the capillary rise h, Jurin's law is obtained:

$$h = \frac{2\gamma \cos\theta}{\rho g r}$$

Jurin's law assumes a constant liquid–solid contact perimeter. However, as shown in Fig. 3b, the wetted perimeter in the BCC structure varies periodically with the liquid position within the unit cell, exhibiting local maxima and minima. Consequently, the corresponding adhesive force is also position-dependent, and the resulting force balance yields an implicit expression for the capillary rise.

As a result, the force balance in the BCC structure cannot be described by a single closed-form expression such as Jurin's law. Instead, the capillary rise was evaluated by applying the force balance to incremental control volumes extending from the bottom of the lattice ($h = 0$) to the liquid surface.

The vertical adhesive force was averaged over the x–y plane and expressed as a function of the local wetted perimeter $s(h)$ and the contact angle:

$$\langle \bar{F}_{adh} \rangle_{xy} = \gamma \cos\theta \int s(h) ds$$

Finally, an explicit analytical expression can be obtained by assuming that the force balance is established at the central node of the BCC unit cell, where both the wetted perimeter and the normalized adhesive force reach their minimum values. When the diagonal strut forms an angle α with the x–y plane, the wetted perimeter can be approximated as $\pi D/\sin\alpha$, leading to the corresponding adhesive force and an explicit solution for capillary rise in a single BCC column:

$$\langle \bar{F}_{adh} \rangle_{1\times1} = \gamma\cos\theta \frac{\pi D}{\sin\alpha} (1)$$

Method S2. Extension of force-balance model to gradient BCC lattice structures

To extend the force-balance model to gradient-configured BCC lattice structures, we consider a simplified system in which a $1\times1\times N_z$ lattice column is composed of vertically stacked unit cells with different strut diameters. Specifically, the structure is assumed to consist of multiple segments, each containing unit cells with uniform geometric properties but varying between segments. Each segment i is characterized by its own geometric parameters, including the strut diameter D_i, angle α_i, width L_1, depth L_2, and relative density ϕ_i.

In contrast to uniform lattices, where a single representative unit cell is sufficient to describe the system, the gradient structure exhibits spatial variations in geometry along the vertical direction. Therefore, both the adhesive force and gravitational force must be evaluated in a segment-wise manner.

Accordingly, the adhesive force as a function of height can be expressed in a piecewise form as follows:

$$\langle \bar{F}_{adh} \rangle_{1\times1,i} = \gamma\cos\theta \frac{\pi D_i}{\sin\alpha_i}$$

Following this, the adhesive force as a function of height can be expressed in a piecewise form:

$$\langle \bar{F}_{adh} \rangle_{1\times1}(h) = \begin{cases} \langle \bar{F}_{adh} \rangle_{1\times1,1}, & (0 \le h \le H_1) \\ \langle \bar{F}_{adh} \rangle_{1\times1,2}, & (H_1 \le h \le H_1 + H_2) \\ \langle \bar{F}_{adh} \rangle_{1\times1,3}, & (H_1 + H_2 \le h \le H_1 + H_2 + H_3) \end{cases}$$

The gravitational force is determined by the cumulative weight of the liquid contained in the structure. Defining the effective liquid cross-sectional area of each segment as:

$$A_i = L_1 L_2 (1 - \phi_i)$$

the gravitational force is expressed as:

$$F_g(h) = \begin{cases} \rho g A_1 h, & (0 \le h \le H_1) \\ \rho g[A_1 H_1 + A_2(h - H_1)], & (H_1 \le h \le H_1 + H_2) \\ \rho g[A_1 H_1 + A_2 H_2 + A_3(h - H_1 - H_2)], & (H_1 + H_2 \le h \le H_1 + H_2 + H_3) \end{cases}$$

This formulation accounts for both the liquid in previously filled segments and the partially filled volume in the current segment. The final capillary height is determined by identifying the segment in which the following condition is first satisfied:

$$\langle \bar{F}_{adh} \rangle_{1\times1,i} = F_g(h)$$

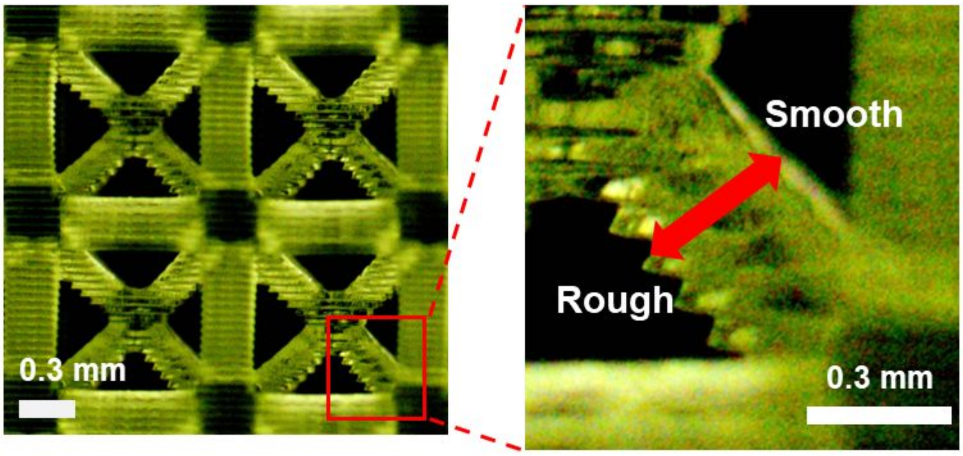

Figure S1. Optical images of a BCC lattice structure indicating smooth and rough surfaces.

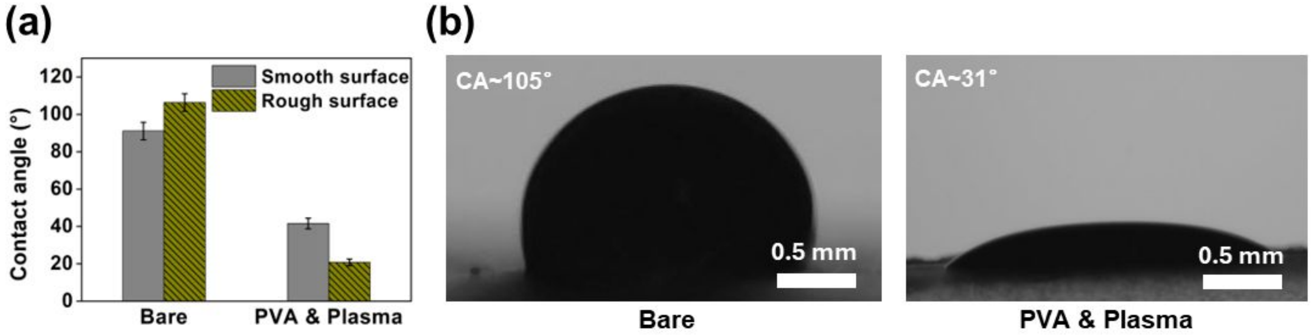

Figure S2. Contact angles on bare and coated rough surfaces. (a) Static contact angles measured on bare and PVA-coated/plasma-treated surfaces, comparing smooth and rough morphologies. (b) Contact angle images of methylene blue on rough surfaces of bare and coated (PVA & plasma treated) samples.

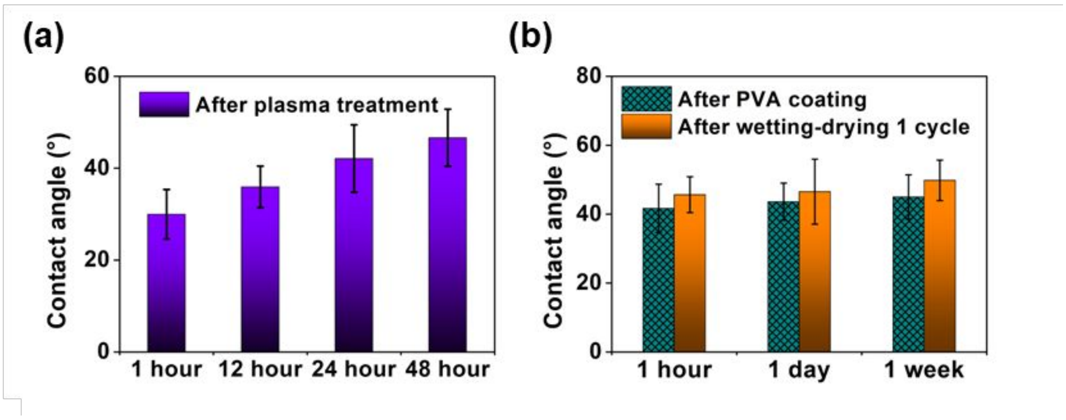

Figure S3. Time-dependent variations in static contact angle on smooth surfaces following surface treatments. (a) Static contact angles measured on plasma-treated surfaces at 1, 12, 24, and 48 h. (b) Contact angles measured after PVA coating and after one wetting–drying cycle at 1 h, 1 day, and 1 week

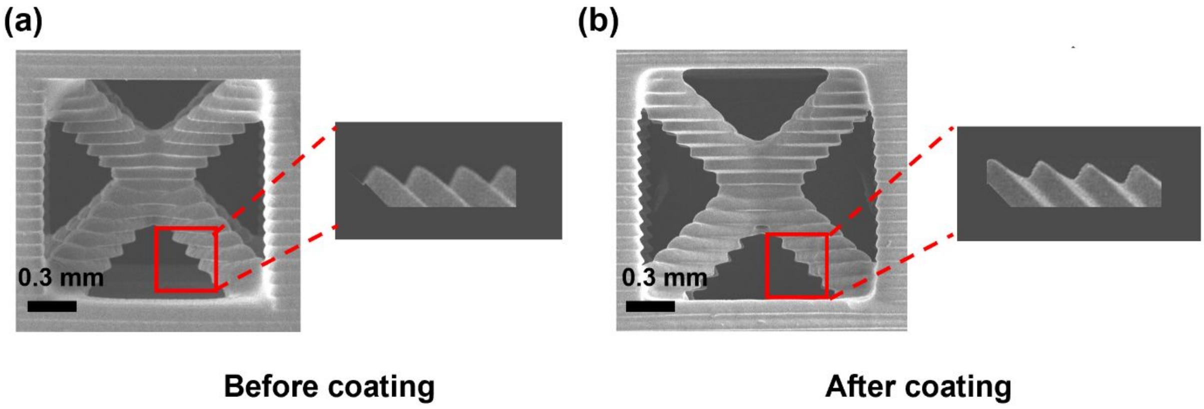

Figure S4. SEM images of the BCC structure showing rough surface features (a) before and (b) after PVA coating.

Surface Condition	R_a	R_q
**Bare**	9.17 ± 0.66	30.0 ± 0.69
**PVA + Plasma**	9.38 ± 1.00	30.9 ± 1.59

Table S1. Surface roughness parameters of the printed samples before and after surface treatment.

Surface	$\theta_{Advancing}$ (°)	$\theta_{Receding}$ (°)	$\Delta\theta$ (°)
**Smooth**	48.6 ± 6.9	15.7 ± 4.6	32.9 ± 8.3
**Rough**	53.6 ± 8.4	8.3 ± 3.2	45.3 ± 9.0

Table S2. Advancing and receding contact angles and contact angle hysteresis (Δθ) measured on smooth and rough printed surfaces.

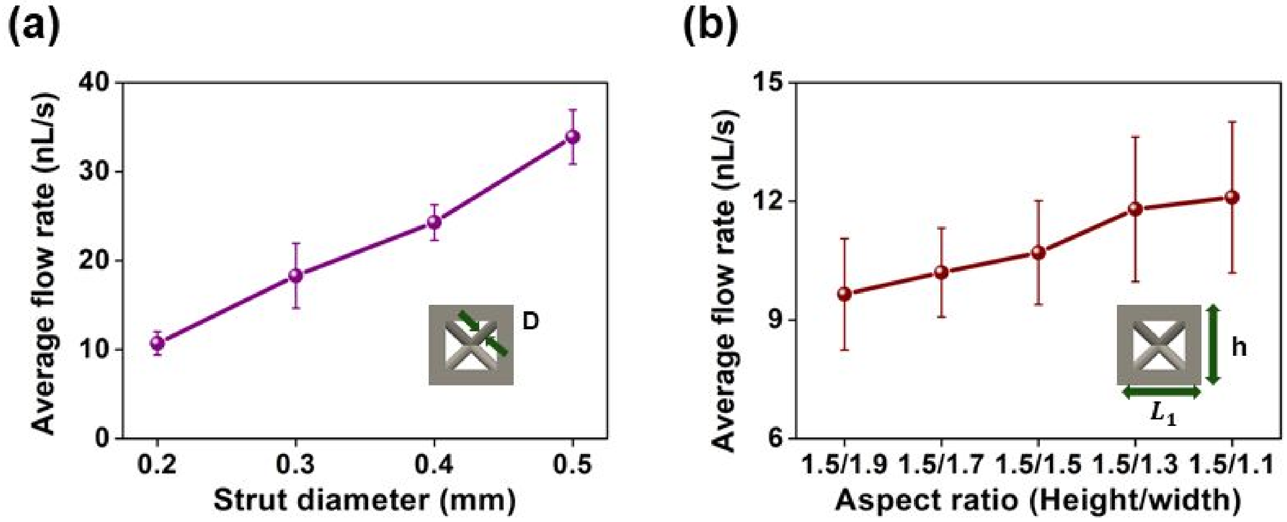

Figure S5. Average capillary-driven flow rates as functions of (a) strut diameter and (b) aspect ratio.

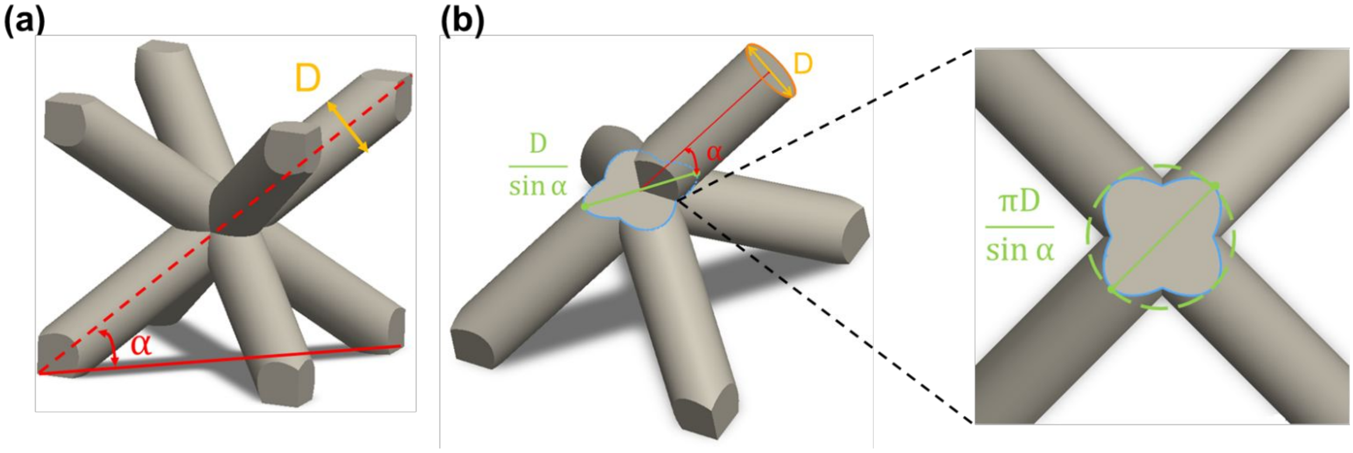

Figure S6. Geometric model for evaluating the wetted perimeter at the unit-cell center. (a) Schematic of a BCC unit cell without frame showing the definition of strut diameter (D) and intersection angle (α). (b) Approximation of the wetted perimeter by a circumscribing circle around the projected diagonal strut cross-sections, yielding an effective perimeter of $\pi D/\sin\alpha$.

Parameter	Value	Approximated perimeter (mm)	Actual perimeter (mm)	Relative error (%)
**Strut diameter (mm)**	0.2	1.09	1.04	4.81
	0.3	1.63	1.57	3.82
	0.4	2.18	2.09	4.31
	0.5	2.72	2.61	4.21
**Aspect ratio (Height/Width)**	1.5/1.1	0.960	0.949	1.16
	1.5/1.3	1.01	0.969	4.23
	1.5/1.5	1.09	1.04	4.81
	1.5/1.7	1.17	1.14	2.63
	1.5/1.9	1.27	1.31	3.05

Table S3. Comparison of approximated and actual wetted perimeters for different strut diameters and aspect ratios.

Lattice size (n × n)	Intercellular contact region $\mathbf{i_n = 2n(n-1)}$	Outer boundary region $\mathbf{o_n = 4n}$	inner / outer Ratio $\mathbf{R_n = \frac{i_n}{o_n} = \frac{\boldsymbol{n}-1}{2}}$
**1 × 1**	0	4	0.00
**2 × 2**	4	8	0.50
**3 × 3**	12	12	1.00
**4 × 4**	24	16	1.50

Table S4. Number of intercellular contact regions and outer boundary regions as a function of lattice size (n × n).

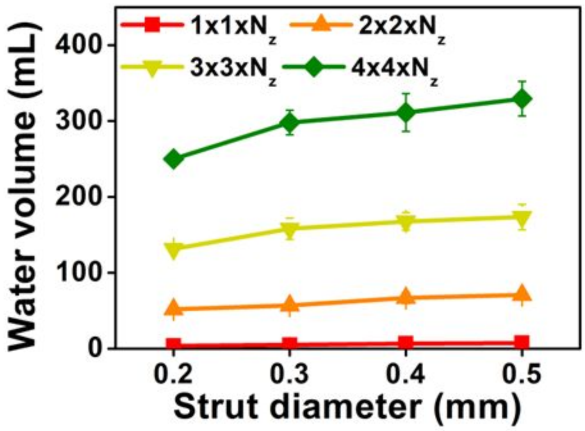

Figure S7. Water volume as a function of strut diameter for different BCC lattices (1 × 1 × N_z to 4 × 4 × N_z)

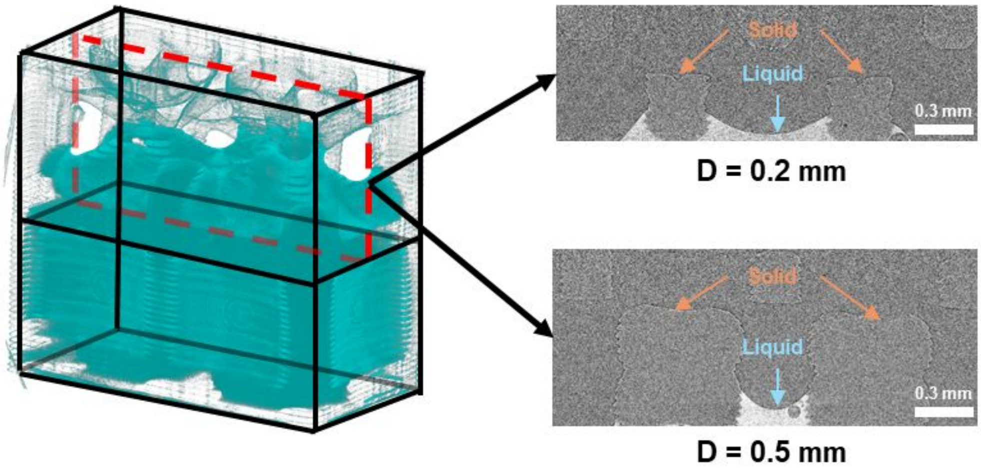

Figure S8. 3D synchrotron X-ray images of the radius of curvature in $2{\times}2{\times}N_z$ BCC lattices.

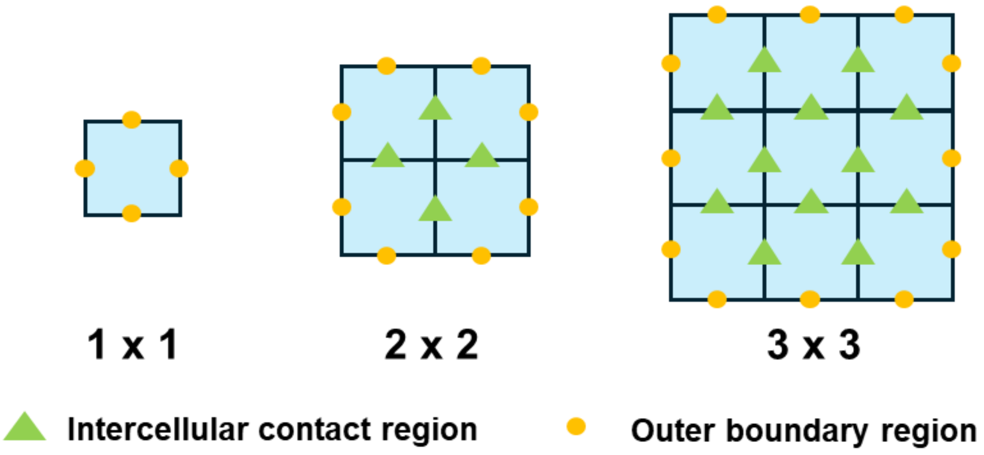

Figure S9. Schematic illustration of intercellular contact regions and outer boundary regions in n × n lattice arrays.

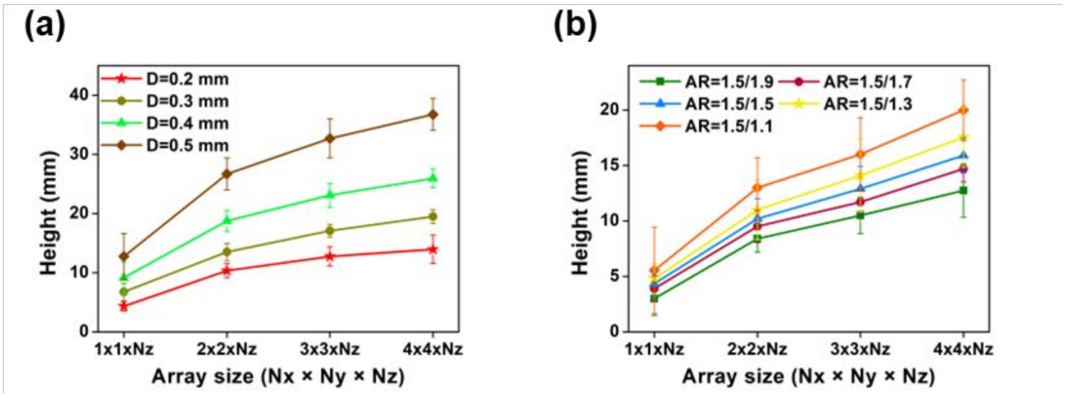

Figure S10. Maximum capillary rise height as a function of cell array size. (a) BCC lattice structures with different strut diameters (D = 0.2, 0.3, 0.4, and 0.5 mm), and (b) structures with varying aspect ratios (1.5/1.9 to 1.5/1.1), showing that the increase in capillary rise height becomes progressively less steep as the array size increases.

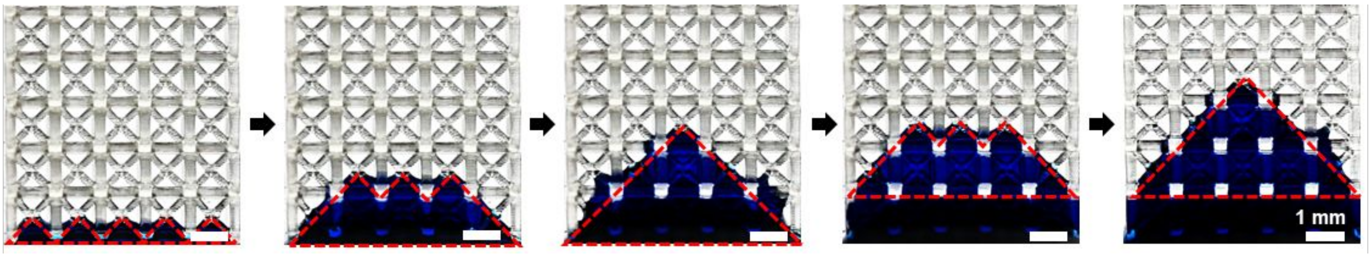

Figure S11. Optical images of BCC lattice structures showing triangular liquid rise patterns.

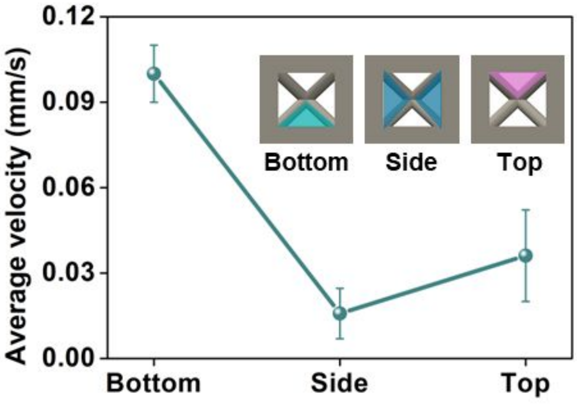

Figure S12 Early-stage average capillary rise velocity at different unit-cell positions (bottom, side, and top).

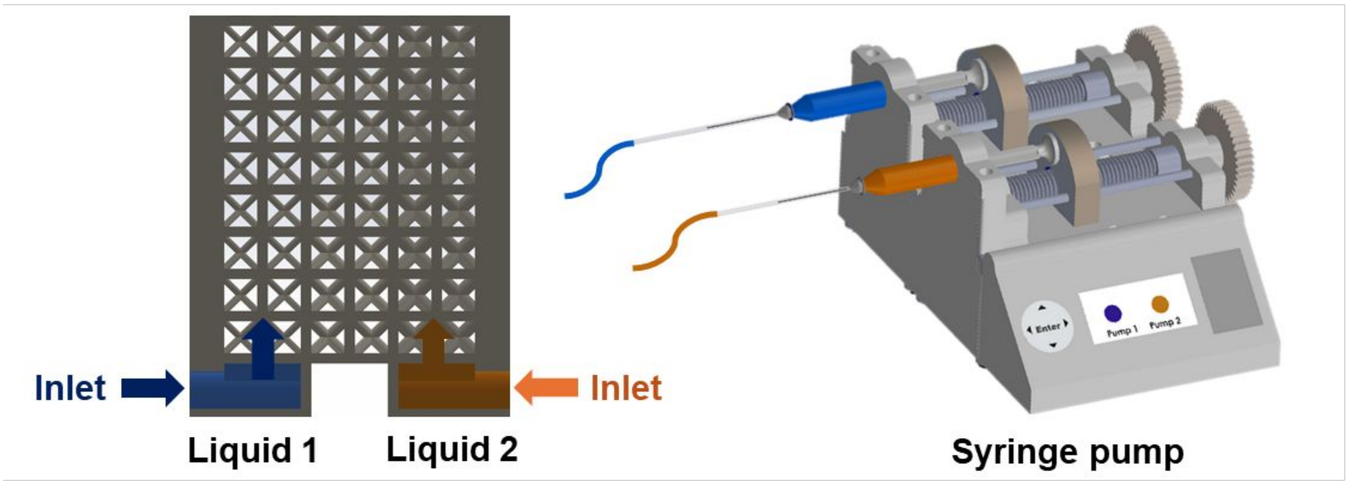

Figure S13. Experimental setup schematic for visualizing the interfacial zone in a BCC lattice.

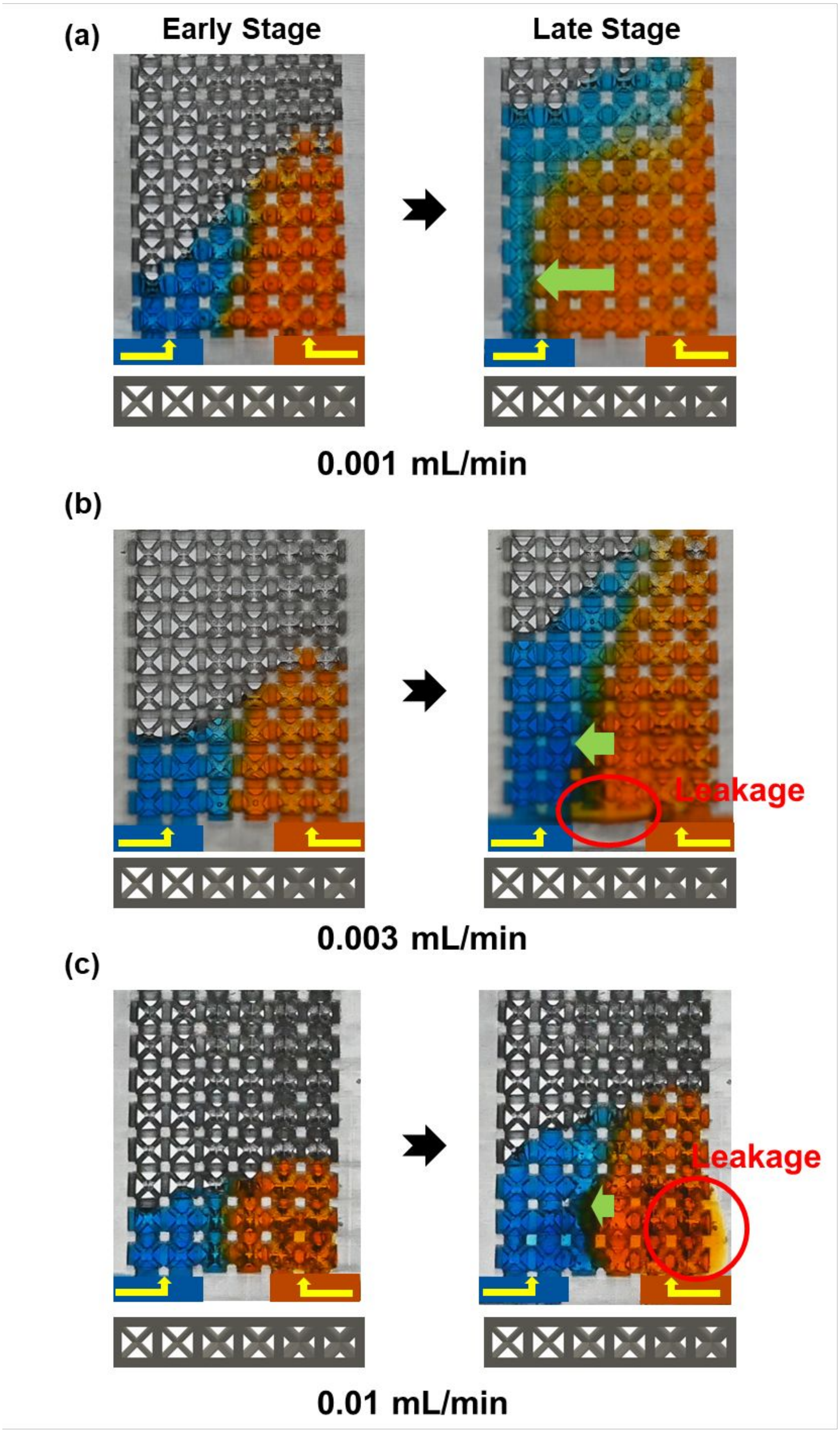

Figure S14. Effect of injection flow rate on interfacial propagation and leakage behavior in asymmetric BCC lattice structures (0.2–0.3–0.4 mm struts) at flow rates of (a) 0.001 mL/min, (b) 0.003 mL/min, and (c) 0.01 mL/min.
